# White matter hyperintensity shape and location feature analysis on brain MRI; proof of principle study in patients with diabetes

**DOI:** 10.1038/s41598-018-20084-y

**Published:** 2018-01-30

**Authors:** Jeroen de Bresser, Hugo J. Kuijf, Karlijn Zaanen, Max A. Viergever, Jeroen Hendrikse, Geert Jan Biessels, Utrecht Vascular Cognitive Impairment Study Group, A. Algra, E. van den Berg, W. Bouvy, M. Brundel, S. Heringa, L. J. Kappelle, A. Leemans, P. R. Luijten, W. P. Th. M. Mali, G. E. H. M. Rutten, K. L. Vincken, J. Zwanenburg

**Affiliations:** 1 Department of Radiology, University Medical Center Utrecht, Utrecht, The Netherlands; 2 Department of Radiology, Leiden University Medical Center, Leiden, The Netherlands; 3 Image Sciences Institute, University Medical Center Utrecht, Utrecht, The Netherlands; 4 Department of Neurology and Neurosurgery, Brain Center Rudolf Magnus, University Medical Center Utrecht, Utrecht, The Netherlands; 5 Julius Center for Health Sciences and Primary Care, University Medical Center Utrecht, Utrecht, The Netherlands

## Abstract

Cerebral small vessel disease is a heterogeneous disease in which various underlying etiologies can lead to different types of white matter hyperintensities (WMH). WMH shape features might aid in distinguishing these different types. In this proof of principle study in patients with type 2 diabetes mellitus (T2DM), we present a novel approach to assess WMH using shape features. Our algorithm determines WMH volume and different WMH shape and location features on 3T MRI scans. These features were compared between patients with T2DM (n = 60) and a matched control group (n = 54). Although a more traditional marker (WMH volume) was not significantly different between groups (natural log transformed Beta (95% CI): 0.07 (−0.11↔0.24)), patients with T2DM showed a larger number of non-punctuate WMH (median (10^th^–90^th^ percentile), patients: 40 lesions per person (16–86); controls: 26 (5–58)) and a different shape (eccentricity) of punctuate deep WMH (Beta (95% CI): 0.40 (0.23↔0.58)) compared to controls. In conclusion, our algorithm identified WMH features that are not part of traditional WMH assessment, but showed to be distinguishing features between patients with T2DM and controls. Future studies could address these features to further unravel the etiology and functional impact of WMH.

## Introduction

White matter hyperintensities (WMH) of presumed vascular origin are a key manifestation of cerebral small vessel disease (SVD) on MRI^1^. SVD is a heterogeneous disease in which various underlying etiologies can lead to different types of WMH^2^. Specific WMH features may distinguish amongst these different types. The WMH burden is currently mainly expressed in terms of volume^1^, but this parameter clearly lacks discriminating potential with regard to etiology. In contrast, certain location and shape features of WMH of presumed vascular origin do provide etiological and even prognostic information. For example, cerebral amyloid angiopathy (CAA) is associated with a more posterior WMH location and Cerebral Autosomal Dominant Arteriopathy with Subcortical Infarcts and Leukoencephalopathy (CADASIL) is associated with a more temporal WMH location^3–5^. WMH in temporal and posterior lobes are also related to an increased risk of dementia^6^. WMH shape and location features may also serve to differentiate WMH of presumed vascular origin from other causes of WMH, such as multiple sclerosis^7^. Such features (e.g. oval shape and involvement of U fibers in multiple sclerosis) are used to establish the most likely etiology of WMH in clinical practice, mostly with visual assessment. However, this can be challenging and many WMH features are not readily visually perceivable. Automated detection and segmentation of WMH is preferred, but also comes with technical challenges. These challenges include partial volume effects especially for small WMH and reproducibility of measurements across MRI field strength, sequence and across variations in sequence parameters^8^.

Most previous studies on WMH have examined WMH volume, sometimes also addressing WMH location (e.g.^9–11^). To the best of our knowledge, WMH shape features have not been explored in depth. To study WMH shape we therefore modified an algorithm that distinguishes different shape features, which was previously used for other medical (for example in lung nodules) and non-medical applications^12,13^. We aimed to apply this algorithm to (1) assess different WMH shape features, (2) determine which features have the best test characteristics for the assessment of WMH shape, and (3) apply this WMH shape feature in a proof of principle study in patients with type 2 diabetes mellitus (T2DM; a condition known to be associated with an increased burden of SVD^14^) and a matched control group.

## Materials and Methods

### Image analysis of WMH features

#### Image preprocessing

Fluid attenuated inversion recovery (FLAIR) images are used for manual segmentation of WMH. This manual segmentation was performed blinded for patient status. To minimize the staircase effect of voxels across slices and to approximate the 3D shape better, a mesh is created of the manual segmentations with the marching cubes algorithm^15^. From the meshes, individual WMH are determined based on connectivity of the neighboring 26 voxels (3D connectivity). Then, WMH consisting of less than five voxels (<0.014 ml) are excluded, because shape analysis cannot be accurately performed on these small lesions. The resulting meshes of the manual segmentations are used as input for the shape and location analysis and to calculate WMH volume.

#### WMH shape features

The WMH shape features that were determined are divided in area based (surface area), dimension/volume based (eccentricity and three measures of compactness) and complex features (fractal dimensions, shape index and curvedness)^13^. These features are calculated in 3D on the meshes of the manual segmentations as follows.

Surface area is calculated from the mesh and corrected for intracranial volume.

Eccentricity is defined as:

1 $$Eccentricity=\frac{diamete{r}_{max}}{diamete{r}_{min}}$$

In this definition ‘diameter_max_’ denotes the largest diameter of the lesion in 3D and ‘diameter_min_’ denotes the smallest diameter of the lesion in 3D orthogonal to ‘diameter_max_’^13^.

The three measures of compactness are defined as^13^:

2 $$Compactness1=\frac{volum{e}^{2}}{are{a}^{3}}$$

3 $$Compactness2=\frac{volume}{di{m}_{x}di{m}_{y}di{m}_{z}}$$

4 $$Compactness3=\frac{volume}{di{m}_{max}^{3}}$$

In these definitions ‘volume’ denotes the volume calculated from the mesh, ‘area’ is the surface area, ‘dim_x_’, ‘dim_y_’ and ‘dim_z_’ are the diameters along the specific axis and ‘dim_max_’ is the maximum diameter along the x, y or z axis.

Fractal dimensions are a measure of topological complexity and are calculated by a box counting method^12^.

Shape index and curvedness values are calculated for all voxels in a lesion and the median of the calculated values was taken to describe a lesion^13^. Shape index and curvedness are defined as:

5 $$Shape\,index=\frac{2}{\pi }{\tan }^{-1}\frac{{k}_{1}+{k}_{2}}{{k}_{1}-{k}_{2}}$$

6 $$Curvedness=\sqrt{{k}_{1}^{2}+{k}_{2}^{2}}$$

In these definitions ‘k_1_’ and ‘k_2_’ are the principal curvatures calculated using the first and second order derivative of the image blurred with a Gaussian filter with scale σ = 1 voxel^13^.

#### WMH location features

To determine location features, all WMH are transformed to MNI152 atlas space^16^ by registering the FLAIR images to MNI with Elastix and applying the transformation parameters to the WMH^17^. In MNI space, WMH located >1.0 cm from the ventricles and with a maximum diameter of <1.5 cm are automatically determined as punctuate deep WMH. These results were manually checked and in some cases corrected. Manual corrections were performed in a minority of patients and within a maximum of 2 lesions per patient. Corrections were needed mostly for segmentation errors due to for the algorithm ‘unconnected parts’ of the lateral ventricles, which can occur in a parietal and occipital location. In MNI space, the location of individual punctuate deep WMH was determined (frontal, temporal, parietal or occipital lobe) and recorded as a location feature.

WMH not classified as punctuate deep WMH consisted of periventricular and (early) confluent WMH and are analyzed as one group named non-punctuate WMH. The location features of non-punctuate WMH are not determined because these lesions often extend in multiple lobes.

### Subject groups

#### Participants

Patients with T2DM and matched controls were participants from the second Utrecht Diabetic Encephalopathy Study (UDES2) (for details see^18,19^). These participants were included through their general practitioners between April 2010 and June 2011. Inclusion criteria were that participants had to be functionally independent, between 65 and 80 years of age and Dutch-speaking. The diagnosis of T2DM was made if participants had diabetes for at least a year, were receiving treatment or had a fasting blood glucose ≥7.0 mmol/L. Exclusion criteria were a psychiatric or neurological disorder that could influence cognitive functioning, nondisabling stroke in the past 2 years, disabling stroke, major depression, alcohol abuse or indications of dementia (mini-mental state examination score ≤24). Control participants with a fasting blood glucose ≥7.0 mmol/L (n = 3), and participants who had severe artefacts on their brain MRI scans or an inadequate scanning protocol (n = 3) were excluded. The participants included in our study all had WMH (60 patients with T2DM and 54 controls). This study was approved by the medical ethics committee of the University Medical Center Utrecht and carried out in accordance with relevant guidelines and regulations. All participants signed an informed consent form.

Details regarding differences in cognitive performance and findings on regular brain MRI measures have been published previously^20^. In short, patients with T2DM performed slightly worse than control subjects on cognitive testing (mean differences in standardized z scores (95% CI) between patients and controls: information processing speed −0.24 (−0.58 to 0.11), attention and executive functioning −0.21 (−0.50 to 0.09), memory −0.14 (−0.44 to 0.17)), but the differences were not statistically significant. Cerebral gray matter volumes were smaller (effect size 0.6, p = 0.02) and lateral ventricle volumes were larger (effect size 0.7, p = 0.02) in the patients with T2DM compared to the control subjects.

#### MRI scanning protocol

Brain MRI scans for all participants were acquired on a Philips 3T MRI system. A standardized protocol was used consisting of, amongst others, a FLAIR sequence (TR: 11000 ms, TE: 125 ms, TI: 2800 ms, acquisition matrix: 232 × 148, slice thickness: 3 mm, acquired voxel size: 0.99 × 1.28 × 3.00 mm^3^, reconstructed voxel size: 0.96 × 0.96 × 3.00 mm^3^), a T1 inversion recovery (IR) sequence (TR: 4416 ms, TE: 15 ms, TI: 400 ms, acquisition matrix: 232 × 185, slice thickness: 3 mm, acquired voxel size: 0.99 × 1.02 × 3.00 mm^3^, reconstructed voxel size: 0.96 × 0.96 × 3.00 mm^3^) and a 3D T1-weighted sequence (TR: 7.9 ms, TE: 4.5 ms, acquisition matrix: 256 × 232, acquired voxel size: 1.00 × 1.00 × 1.00 mm^3^, reconstructed voxel size: 1.00 × 1.00 × 1.00 mm^3^).

#### Other MRI measures

Presence of cerebral lacunar infarcts and large vessel infarcts (>1.5 cm) was rated visually on the FLAIR and 3D T1-weighted MRI images. Gray and white matter volumes were determined automatically on the 3D T1-weighted images by using FreeSurfer (http://surfer.nmr.mgh.harvard.edu;^21^). Intracranial volumes were manually segmented on the T1 IR images^20^. Gray and white matter volume were expressed as a percentage of intracranial volume.

### Statistical analysis

WMH shape features (surface area, eccentricity, three measures of compactness, fractal dimensions, shape index and curvedness) were calculated for the WMH in all subjects. For these WMH features mean, minimum, maximum and skewness were calculated per lesion. Kolmogorov-Smirnov tests were performed to test for non-normal distribution.

Regarding the participant groups, differences in characteristics between the patients with T2DM and controls were assessed with T-tests for continuous variables, χ^2^ tests for proportions and Mann-Whitney U Tests for non-parametric data. WMH volumes and numbers were natural log transformed because of non-normal distribution (Kolmogorov-Smirnov; p < 0.05). To retain the direction of effect, the WMH volumes were first scaled to a range above 1 (multiplication by 10000). Between-group differences in WMH volume (as a percentage of intracranial volume), number and shape (median eccentricity) were assessed with linear regression analyses adjusted for age and sex. These differences were analyzed separately for all WMH, non-punctuate WMH and punctuate deep WMH. To assess between-group differences in location features (frontal, temporal, parietal and occipital) of punctuate deep WMH, χ^2^ tests were performed on the percentages of WMH per location. Between-group differences in punctuate deep WMH shape per location were analyzed with Mann Whitney U tests.

As a secondary analysis within the group of patients with T2DM, the association of features of all WMH (volume, number and shape (eccentricity)) with white matter volume, gray matter volume and diabetes duration was assessed with linear regression analyses adjusted for age and sex (and additionally for intracranial volume for WMH volumes).

### Data availability

Anonymized imaging data are publicly available from the Utrecht Vascular Cognitive Impairment Study Group (http://www.isi.uu.nl/Research/Databases/WMHShape/). Questions can be addressed to Manja Litjens (MLitjens@umcutrecht.nl) of the Utrecht Vascular Cognitive Impairment Study Group Data Access Committee.

## Results

### WMH shape features

An overview of the different WMH shape features for both subject groups combined is shown in Table 1. All WMH features had a non-normal distribution (Kolmogorov-Smirnov; p < 0.05). In this respect surface area shows the largest skewness (13.82) compared to the other WMH features (≤2.20). Surface area, all compactness measures, eccentricity and fractal dimensions show a floor effect (the minimum measured value is close to or similar to the smallest possible value of these features). Compactness2 and compactness3 show a ceiling effect (the maximum measured value is close to or similar to the largest possible value of these features). This implies that variance in WMH shape might not be adequately measured by these features. The output of the complex WMH features (fractal dimensions, shape index and curvedness) proved to be difficult to comprehend and link to visual observations of WMH shape.

**Table 1:** Overview of different WMH shape features per lesion.

	Mean	Minimum	Maximum	Skewness	Kolmogorov-Smirnov
**Area based**					
Surface area^†^	0.01	0.00	1.09	13.82	p < 0.001
**Dimension/volume based**					
Eccentricity	2.52	1.00	8.97	1.57	p < 0.001
Compactness1	0.0039	0.0001	0.0073	−0.10	p < 0.001
Compactness2	0.52	0.03	1.00	0.25	p < 0.001
Compactness3	0.20	0.00	1.00	1.14	p < 0.001
**Complex features**					
Fractal dimensions	1.37	0.00	2.62	−0.51	p < 0.001
Shape index	−0.64	−0.73	−0.45	0.86	p < 0.001
Curvedness	2.30	0.63	7.71	2.20	p < 0.001

Mean, minimum, maximum, skewness and output of the Kolmogorov-Smirnov test are shown for different WMH shape features. These values were calculated per lesion on the combined patient and control group.

^†^Corrected for intracranial volume.

**Figure 1:**
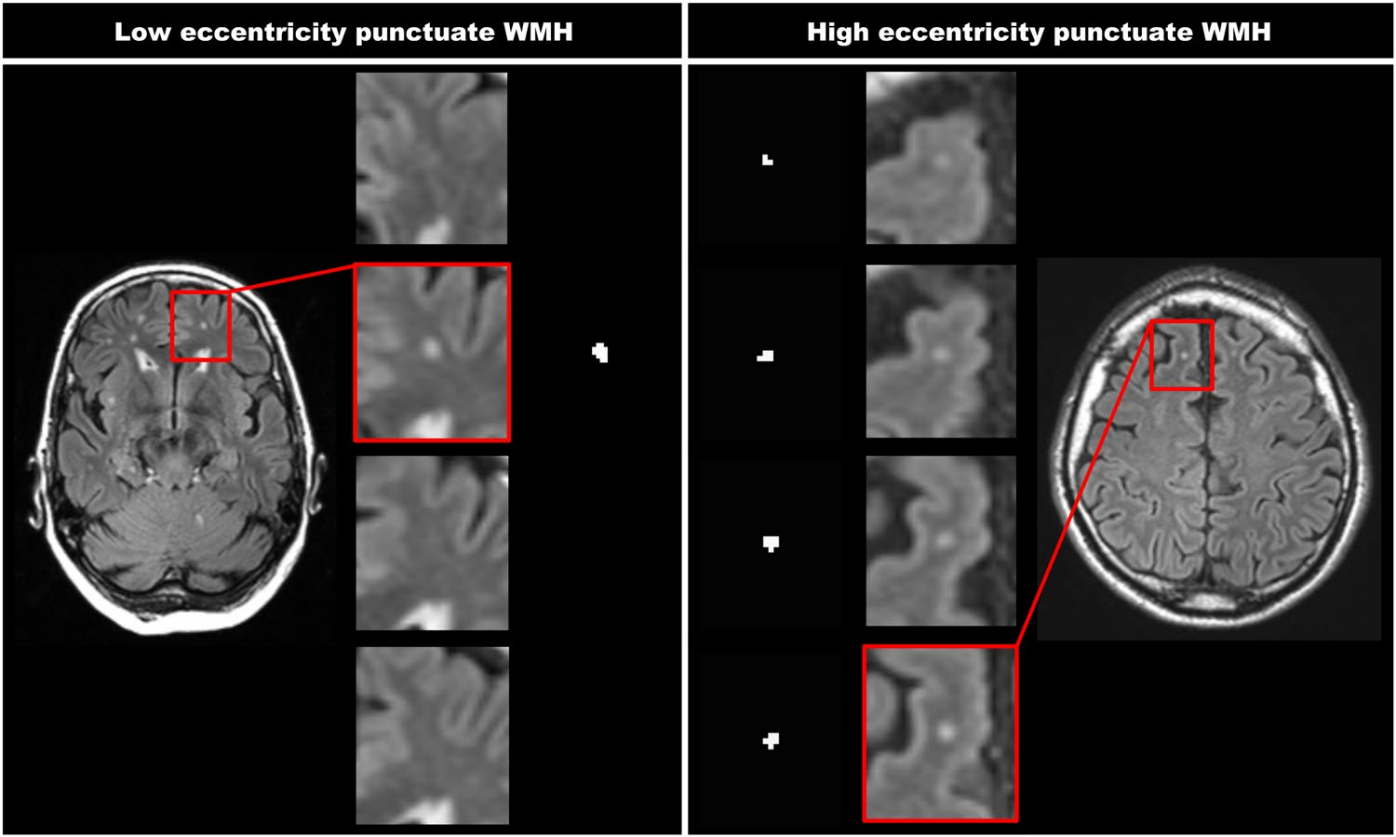
Two WMH with a different eccentricity value. This figure represents two WMH that have a different eccentricity value. The shown FLAIR images have a voxel size of 0.96 × 0.96 × 3.00 mm^3^. The left panels show a punctuate deep WMH with a low eccentricity of 1.0 (close to spherical), which is seen in only one slice. The right panels show a punctuate deep WMH with a high eccentricity of 4.2 (strongly ellipsoidal), which is caused by the lesion extending in multiple slices. As can be appreciated, this difference in WMH shape can also be perceived visually.

Eccentricity was chosen to test as a WMH shape feature for the between group comparisons, because it is translation-, scale- and rotation-invariant, has a relatively small skewness, does not show a ceiling effect in our measurements, has a limited floor effect (a perfect sphere) and is easy to comprehend and link to visual observations of WMH shape. An example of the WMH shape feature eccentricity for a punctuate deep WMH is shown in Fig. 1. A low eccentricity means close to spherical and a high eccentricity means strongly ellipsoidal. As can be appreciated from the figure, this difference in WMH shape can also be perceived visually.

### WMH features in the subject groups

The characteristics of the group with T2DM (mean age (range): 71 years (65–80)) and the control group (71 years (66–80)) are shown in Table 2. Compared to controls, patients showed a smaller gray matter volume (p = 0.009). White matter volume, lacunar infarcts and large vessel infarcts showed no between group differences (p > 0.05).

**Table 2:** Characteristics of the subject groups.

	Patients with T2DM (n = 60)	Control participants (n = 54)	p-values
**Characteristics**			
Men (%)	35 (58)	32 (59)	0.920
Age (years)	71.0 ± 4.3	71.2 ± 4.7	0.794
Mean arterial pressure (mmHg)^†^	103 ± 10	102 ± 11	0.613
HbA1c (%)^‡^	6.7 (5.9–7.8)	5.6 (5.3–6.2)	<0.001
Diabetes duration (years)	10.6 ± 8.7	—	—
**MRI**			
Lacunar infarcts (%)	20 (33)	17 (31)	0.833
Large vessel infarcts (%)	4 (7)	1 (2)	0.203
Gray matter volume (%ICV)	38.0 ± 2.3	39.1 ± 2.1	0.009
White matter volume (%ICV)	29.8 ± 2.4	30.2 ± 3.0	0.447

Data are n (percentage), mean ± SD or median (10^th^–90^th^ percentile).

^†^Systolic and diastolic blood pressure were measured on three different time points. The mean arterial pressure was calculated from the averaged systolic and diastolic blood pressure.

^‡^Determined with standardized laboratory testing.

T2DM: type 2 diabetes mellitus. %ICV: percentage of intracranial volume.

WMH volume, number and shape (eccentricity) for all WMH, non-punctuate WMH (periventricular and (early) confluent WMH) and punctuate deep WMH are shown in Table 3; differences between patients and controls are shown as regression B coefficients (95% CI) and regression Beta coefficients (95% CI). Total WMH volume was similar in patients with T2DM and controls (natural log transformed (NL) Beta (95% CI): 0.07 (−0.11↔0.24)), but the number of WMH was larger (median (10^th^–90^th^ percentile), patients: 55 (17–123); controls: 40 (9–90)) and eccentricity of WMH was higher (Beta (95% CI): 0.34 (0.16↔0.51)).

**Table 3:** WMH features per subject.

	Patients with T2DM	Control participants	Differences between patients and controls^†^	
			B (95% CI)	Beta (95% CI)
**All WMH (60 patients; 54 controls)**				
Volume (%ICV)	0.20 (0.04–0.86)	0.19 (0.03–0.89)	0.16 (−0.27↔0.58)	0.07 (−0.11↔0.24)
Number	55 (17–123)	40 (9–90)	0.42 (0.12↔0.71)*	0.25 (0.07↔0.43)*
Shape (eccentricity)^‡^	2.36 ± 0.30	2.10 ± 0.40	0.25 (0.12↔0.38)*	0.34 (0.16↔0.51)*
**Non-punctuate WMH (60 patients; 54 controls)**				
Volume (%ICV)	0.19 (0.04–0.82)	0.18 (0.03–0.80)	0.27 (−0.21↔0.74)	0.10 (−0.08↔0.28)
Number	40 (16–86)	26 (5–58)	0.62 (0.33↔0.92)*	0.37 (0.19↔0.54)*
Shape (eccentricity)^‡^	2.48 ± 0.36	2.37 ± 0.61	0.10 (−0.08↔0.28)	0.10 (−0.08↔0.29)
**Punctuate deep WMH (56 patients; 52 controls)**				
Volume (%ICV)	0.015 (0.001–0.061)	0.017 (0.003–0.102)	−0.28 (−0.80↔0.24)	−0.10 (−0.29↔0.09)
Number	12 (2–32)	12 (2–43)	−0.11 (−0.52↔0.30)	−0.05 (−0.24↔0.14)
Shape (eccentricity)^‡^	2.06 ± 0.35	1.79 ± 0.25	0.27 (0.15↔0.39)*	0.40 (0.23↔0.58)*

Lesion volume (median (10^th^–90^th^ percentile)), number (median (10^th^–90^th^ percentile)) and shape (mean ± SD) of WMH are shown for patients and controls. These values are shown separately for all WMH, non-punctuate WMH (periventricular and (early) confluent WMH) and punctuate deep WMH. Differences between patients and controls are regression B coefficients (95% CI) and regression Beta coefficients (95% CI); both adjusted for age and sex.

^†^Volume and number represent natural log transformed values. For volumes, values were first scaled to a range above 1 (multiplication by 10000) to retain the direction of effect. Within groups eccentricity showed a normal distribution (Kolmogorov-Smirnov; p > 0.05).

^‡^Shape per subject represents the median eccentricity value of individual WMH.

^*^p < 0.01

T2DM: type 2 diabetes mellitus. WMH: white matter hyperintensities. %ICV: percentage of intracranial volume.

For non-punctuate WMH the eccentricity (Beta (95% CI): 0.10 (−0.08↔0.29)) and volume (NL Beta (95% CI): 0.10 (−0.08↔0.28)) of WMH was similar in both groups, but patients showed a larger number of WMH (median (10^th^–90^th^ percentile), patients: 40 (16–86); controls: 26 (5–58)). This implicates that patients had more non-punctuate WMH than controls, but the mean volume per lesion was smaller.

**Figure 2:**
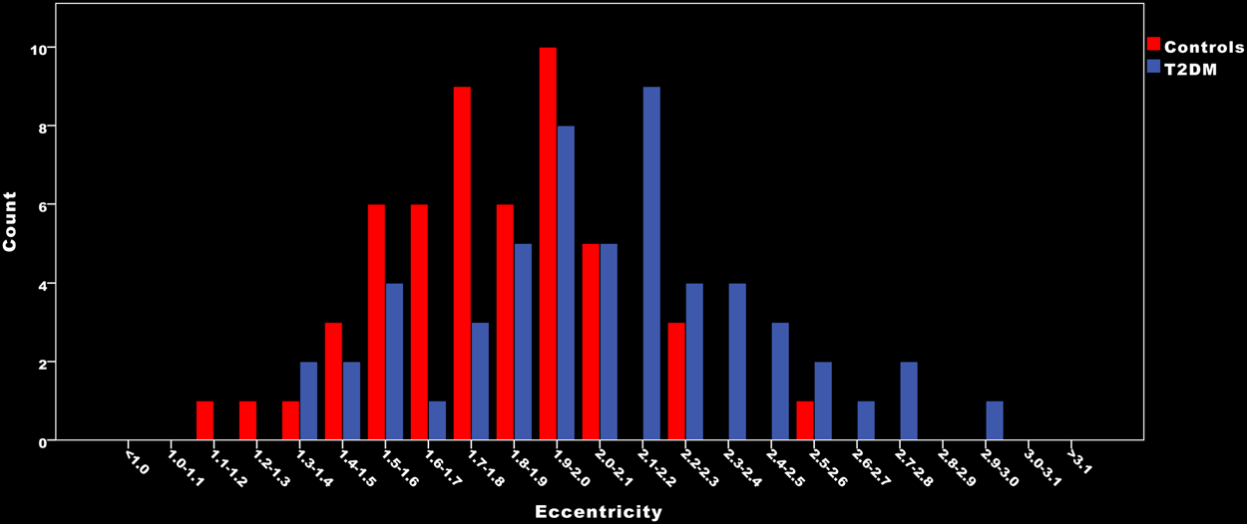
Median eccentricity of punctuate deep WMH per participant.

For punctuate deep WMH the volume (NL Beta (95% CI): −0.10 (−0.29↔0.09)) and number (median (10^th^–90^th^ percentile), patients: 12 (2–32); controls: 12 (2–43)) of WMH were similar between patients and controls, but WMH in patients had a higher eccentricity (Beta (95% CI): 0.40 (0.23↔0.58)). These group differences between the eccentricity of punctuate deep WMH can be observed in Fig. 2.

**Figure 3:**
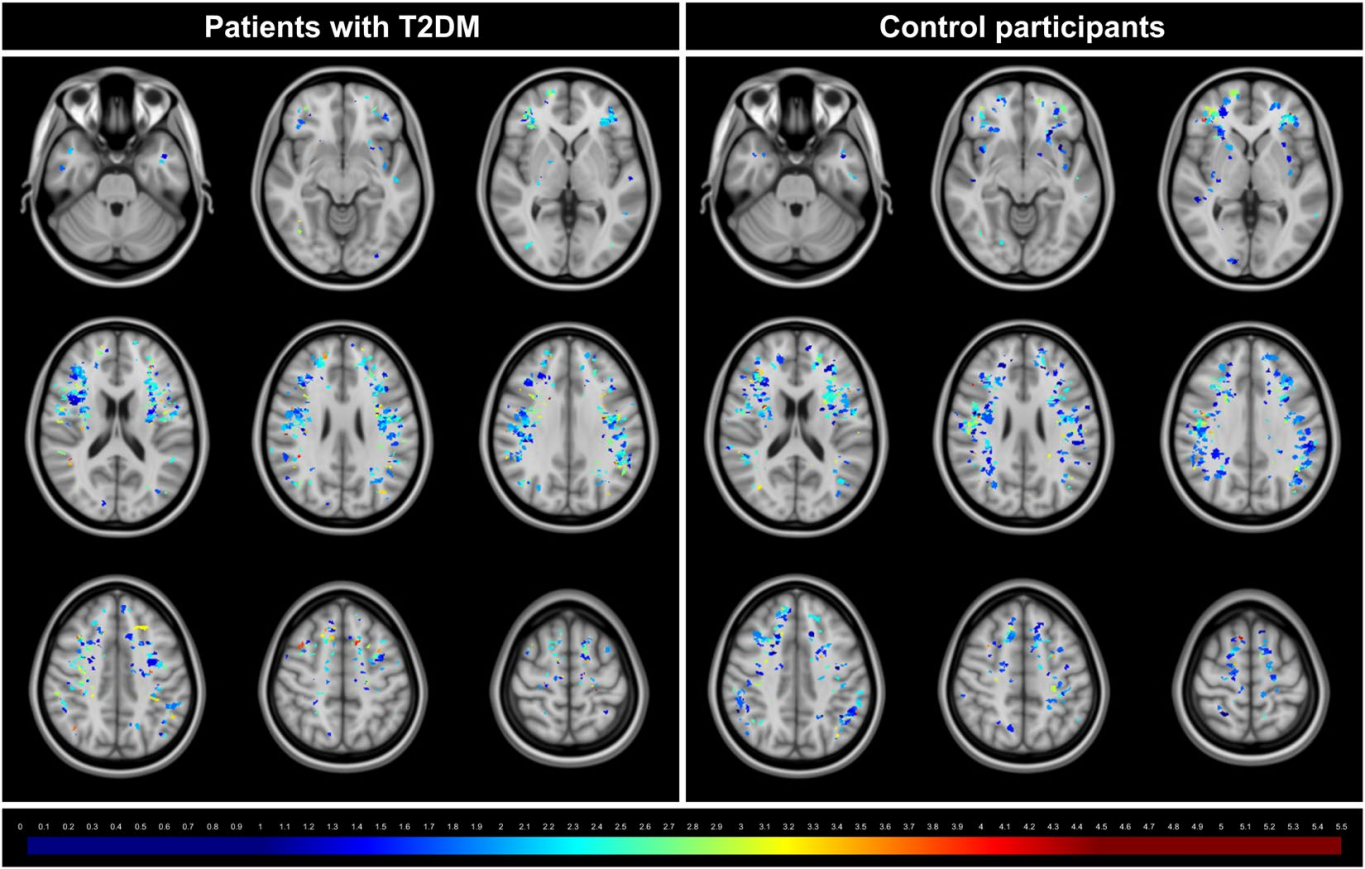
Mean eccentricity maps of the punctuate deep WMH. This figure illustrates mean eccentricity maps of the punctuate deep WMH for the group of patients with type 2 diabetes mellitus (T2DM) as well as for the control group. Each colored voxel represents presence of a WMH on that location in at least one participant and the color itself represents the mean eccentricity of all WMH on that location. The colors range from dark blue (low mean eccentricity) to dark red (high mean eccentricity). This figure illustrates that most punctuate deep WMH were in a frontal and parietal location. It also illustrates that in a frontal and parietal location there are visually less dark blue WMH in the patient group compared to the control group. These maps were obtained by automatic registration of the punctuate deep WMH to MNI152 atlas space^16^. Then, voxels with WMH were assigned to their respective eccentricity value (0 for non-lesion voxels). In both groups the eccentricity values were summed per voxel and divided by the lesion count per voxel, to obtain average eccentricity values per lesion-voxel. Due to minor registration errors some lesions are shown in cortical gray matter on the template image. This had no effect on our statistical analyses, as this template registration was only performed for the current figure.

For punctuate deep WMH it is also possible to determine the number and eccentricity of WMH per lobe (see Table 4). In the patient group a total of 594 WMH were located in a frontal location, 46 in a temporal location, 213 in a parietal location and 13 in an occipital location. The distribution across lobes was similar for patients and controls (all p > 0.05). Compared to controls, the patients had a higher eccentricity of WMH in a frontal and parietal location (p < 0.05). These results are graphically visualized in Fig. 3. This figure shows combined mean eccentricity maps of the punctuate deep WMH for the group of patients with T2DM as well as for the control group. This figure illustrates visually that most punctuate deep WMH were in a frontal and parietal location.

**Table 4:** WMH shape and location features per lesion for punctuate deep WMH.

	Punctuate deep WMH in patients with T2DM (n = 866)	Punctuate deep WMH in control participants (n = 881)	p-values
**Location (number)**			
Frontal	594 (68%)	561 (64%)	0.550
Temporal	46 (5%)	74 (8%)	0.390
Parietal	213 (25%)	234 (27%)	0.747
Occipital	13 (2%)	12 (1%)	0.561
**Shape (eccentricity)**			
Frontal	2.06 (1.40–3.06)	1.81 (1.30–2.64)	<0.001
Temporal	1.86 (1.35–2.96)	1.66 (1.27–2.50)	0.084
Parietal	1.99 (1.38–2.99)	1.81 (1.35–2.75)	0.001
Occipital	2.00 (1.32–2.84)	1.78 (1.33–2.79)	0.769

Location (n (percentage)) and shape (median (10^th^–90^th^ percentile)) are shown per lesion for punctuate deep WMH. For location, χ^2^ tests were performed on the percentages. For shape, Mann Whitney U tests were performed on the eccentricity values. Because of the limited sample size the 17 WMH located in the basal ganglia region and cerebellum are not presented in this table.

T2DM: type 2 diabetes mellitus. WMH: white matter hyperintensities.

Within the group of patients with T2DM, no significant associations were found between white matter volume, gray matter volume and diabetes duration on one side and features of all WMH (volume, number and shape (eccentricity)) on the other side (p > 0.05).

## Discussion

Our algorithm provides assessment of WMH volume, location and shape features, including surface area, eccentricity, different compactness measures, fractal dimensions, shape index and curvedness. Eccentricity was chosen for the proof of principle study because of favorable test characteristics. In this study, patients with T2DM did not differ from controls on traditional WMH measures (total WMH volume), but patients had more non-punctuate WMH and a difference in shape (eccentricity) of punctuate deep WMH compared to controls.

### WMH features

WMH of presumed vascular origin are a key MRI manifestation of SVD^1^. Total WMH volume is the feature of WMH of presumed vascular origin that is studied most frequently (e.g.^22–29^). Few studies have also added some detail on location features (e.g.^9–11^). WMH shape features have, to our knowledge, not been explored in depth. We are also the first to provide assessment of WMH volume, location features as well as shape features. With this approach, we identified WMH features that are not part of a traditional WMH assessment, but proved to differ between patients with T2DM and controls. This study thus shows proof of principle that WMH features provide novel perspectives on cerebral SVD. Importantly, extremes of WMH shape, as identified through the algorithm, can also be perceived visually (see Fig. 1).

### Neuropathology versus MRI of WMH

Neuropathological studies of WMH of presumed vascular origin showed heterogeneous underlying abnormalities^30–32^. The two main pathological types of WMH are abnormal white matter areas (consisting of edema) around widened venules without ischemic changes and arteriopathy with ischemic changes^30,32^. Smooth periventricular WMH on MRI appear to be non-vascular in origin on pathology and are considered normal anatomical structures^32^. On the other hand, punctuate and (early) confluent WMH on MRI showed heterogeneous underlying pathological changes^32^. Confluent WMH are generally considered related to underlying ischemic changes, while punctuate deep WMH are generally considered non ischemic^32^. However, at some point punctuate deep WMH are starting to progress towards ischemic changes/confluency. This can sometimes be observed on brain MRI as a focal area of acute ischemia next to a punctuate deep WMH, which precedes the occurrence of more extensive WMH in the same area^1,2^. Regarding our observations on MRI, possibly the first quantifiable step towards these ischemic changes/confluency is change of punctuate deep WMH to a more ellipsoidal shape (which is the dominant shape characteristic of early confluent WMH).

### Strengths and limitations

The strength of our study is that our algorithm provides assessment of WMH volume, location features as well as shape features. For our proof of principle study we have focused on eccentricity, because of favorable test characteristics (mainly because it is an easy to understand 3D WMH shape feature that is translation-, scale- and rotation-invariant). We have also shown the versatility of our method, because within the same framework also other WMH features can be determined (like surface area, measures of compactness, fractal dimensions, shape index and curvedness)^13^. A limitation of our method could be that it is dependent on accurate segmentations of all WMH. Of note, most automated WMH segmentation methods have a relatively lower accuracy compared to methods for segmentation of other brain structures^33–35^. Automated WMH segmentation methods also have a tendency to undersegment peripherally located punctuate deep WMH. In contrast, voxels that are erroneously segmented as WMH usually only have a limited effect on WMH volume, but could have a larger effect on WMH shape features especially in patients with a low WMH burden. We therefore chose to use the reference standard of manual segmentation of the WMH, but this approach is clearly labor intensive. For future automation of our method, an improvement of current WMH segmentation methods will be crucial to be able to accurately assess all WMH. Another limitation of our study could be the use of 2D multi-slice FLAIR images with anisotropic voxels. The use of 3D FLAIR images with isotropic voxels will further increase the accuracy of WMH segmentations. This will result in a higher precision and accuracy of our WMH shape features. Despite this limitation, we were able to find between group differences with the use of our 2D FLAIR images.

### Future implementations

Shape features of WMH of presumed vascular origin can provide additional information regarding etiology and possibly prognosis^3–6^. The algorithm might also be useful in distinguishing between WMH of presumed vascular origin and WMH of other etiology, for example of demyelinating origin^36^. Our findings show that analysis of WMH shape and location features can identify differences between patient groups that cannot readily be perceived by the naked eye. Of note, our proof of principle study was not designed to identify the exact pathophysiological processes underlying different WMH features. This should be explored in further studies.

## Electronic supplementary material

Database
